# Orthogonal-band-multiplexed offset-QAM optical superchannel generation and coherent detection

**DOI:** 10.1038/srep17891

**Published:** 2015-12-08

**Authors:** Zhennan Zheng, Dan Wang, Xiaoqi Zhu, Xin Lv, Kaiheng Zou, Yixiao Zhu, Fan Zhang, Zhangyuan Chen

**Affiliations:** 1 State Key Laboratory of Advanced Optical Communication Systems and Networks, Peking University, Beijing, 100871, China

## Abstract

Nowadays the Internet not only has fast growing data traffic, but also has a fast growing number of on-line devices. This leads to high demand of capacity and flexibility of the future networks. The conventional Orthogonal Frequency Division Multiplexing (OFDM) and Nyquist pulse shaping signals have the advantage of high spectral efficiency when consisting of superchannels in the Wavelength-Division-Multiplexing (WDM) way. However, they face a cost issue when the spectral granularity of the superchannel is decreased to support more users. This paper proposes for the first time the scheme of Orthogonal-band-multiplexed offset-Quadrature Amplitude Modulation (OBM-OQAM) superchannel. OBM-OQAM superchannel provides large capacity and high spectral efficiency. Furthermore, it has the advantage of offering subbands of variable symbol rate without changing the system configuration. We provide a proof-of-principle demonstration of OBM-OQAM superchannel transmission. In our experiment, 400 Gbps 16 Quadrature Amplitude Modulation (QAM) OBM-OQAM superchannel transmission over 400 km Standard Single Mode Fiber (SSMF) is conducted. The experimental results show that the OBM-OQAM signal has low penalty in multi-band aggregation.

The Internet data traffic continues to grow at a compound annual growth rate of 23% per year^1^. The quantity of user devices connected to IP networks also grows fast. Predicted by ref. 1, it will be nearly three times as high as the global population in 2019. Therefore the future transmission systems should provide larger capacity, and become more flexible to support the growing number of users. Considering the available bandwidth of optical fiber is limited, the transmission system with high spectral efficiency and flexibility becomes more and more important.

Figure 1 shows the scheme of state-of-the-art high spectral efficiency optical superchannel. There are two common ways to increase the spectrum efficiency. One way uses multi-level modulation format such as Quadrature Phase Shift Keying (QPSK) or higher order M-QAMs^2^,^3^,^4^. While the modulation order is not unlimited, higher-order modulation format requires higher Signal to Noise Ratio (SNR) to receive signals under the same Bit Error Rate (BER) target. The other way uses high spectral efficiency modulating technologies such as OFDM or Nyquist pulse shaping. An OFDM signal consists of a set of subcarriers, each one uses rectangular pulse which exhibits a sinc-shaped spectrum. Therefore OFDM subcarriers can be spaced at the baud rate interval without inter-subcarrier-interference. In contrast, Nyquist pulses have rectangular spectra and they overlap in the time domain without inter-symbol interference (ISI). By aggregating multi Nyquist subbands or OFDM signals with high order modulation format in a WDM way, high speed and high spectral efficiency superchannels will be obtained^5^,^6^,^7^,^8^,^9^,^10^, which is shown in Fig. 1(a–c). In ref. 7, 101.7 Tb/s 128QAM-OFDM-WDM signal is generated with the spectral efficiency of 11 b/s/Hz. In ref. 9, 640 Nyquist subbands are composed to reach a data rate of 64 Tb/s and a spectral efficiency of 8 b/s/Hz. Ref. 10 demonstrates 107 Gb/s QPSK OBM-OFDM superchannel, in which orthogonality condition is satisfied between OFDM subbands, but it also needs guard bands to reduce the inter-subcarrier-interference caused by the phase noise between the adjacent carriers. The bandwidth of the guard band is an integral multiple of the subcarrier interval (Fig. 1(c)). In practical application, each subband of such superchannels requires one independent modulator and the corresponding laser. The setup need to be changed if we modify the spectral granularity of the superchannel. We will face a cost issue when decreasing the spectral granularity. For example, if the baud rate of each subband reduces to 1/N while the capacity and bandwidth remains the same, the amount of subbands should be N times larger, which means the system requires N-fold modulators and lasers. ref. 11 demonstrates an all-optical 26 Tb/s, 5 b/s/Hz OFDM signal. In ref. 11, an optical comb generator is used to offer all 75 subcarriers which come from only one laser source, but each subcarrier still requires one independent modulator.

Multicarrier offset quadrature amplitude modulation (MC-OQAM) signal (Fig. 1(d)) also has high spectral efficiency. MC-OQAM was originally proposed as a parallel data transmission scheme in 1967^12^, and was recently brought into coherent optical communication as an alternative that achieves subband spacing equal to the symbol rate^13^,^14^,^15^,^16^. By using root-raised-cosine (RRC) pulse shaping and introducing half-symbol delay alternating between the in-phase and the quadrature component in even and odd subbands, the spectra of adjacent subbands would overlap without resulting in crosstalk. Refs 15 and 16 accomplish generating and detecting electrically orthogonal MC-OQAM signal, where several high spectral efficiency electrical subbands are generated by one signal source, and are modulated to one optical carrier. Especially in ref. 15, a 224 Gb/s PDM-16QAM MC-OQAM real-time transmitter is exhibited. MC-OQAM inherits the performance of the Nyquist pulses^13^,^16^ and offers a new method of composing high spectral efficiency multiband signal. However, limited by the electronic bandwidth bottleneck of the Digital to Analog Converter (DAC), the electrically orthogonal MC-OQAM signal would not occupy a wide bandwidth nor provide high capacity.

This paper proposes the scheme of Orthogonal-band-multiplexed OQAM (OBM-OQAM) superchannel, which is shown in Fig. 1(e). The OBM-OQAM superchannel consists of groups of subbands. Each group is modulated by an electrical MC-OQAM signal, offering equidistant subbands of variable symbol rate and high spectral efficiency. No guard band is required in the superchannel. The receiver can demodulate any subband individually, or several subbands as a whole, which is shown in Fig. 1(f). If the receiver bandwidth is large enough, the full-band receiving of the superchannel can be done also. With the help of the optical frequency comb generator (OFCG), a proof-of-concept experiment is demonstrated, in which a 400 Gb/s 16QAM OBM-OQAM superchannel is transmitted over 400 km SSMF.

## Results

### OBM-OQAM scheme

**Figure 2:**
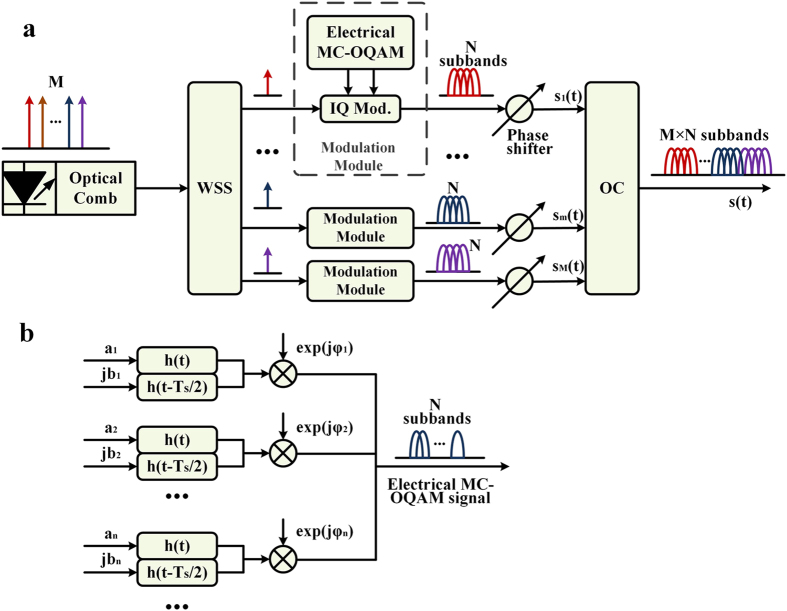
OBM-OQAM transmitter scheme. (**a**) Equidistant optical carriers are split into M branches, each one is modulated by N electrical MC-OQAM subbands. Then all N × M subbands are combined to form an OBM-OQAM superchannel. The adjacent subbands have a constant interval that equals to the symbol rate even though they originate from different brunches. (**b**) Generation of electrical MC-OQAM signal.

Figure 2(a) shows the proposed OBM-OQAM transmitter scheme. The multiple optical carriers are generated by an optical comb which originates from one single laser. After separated by a wavelength-selective switch (WSS), each carrier is modulated by several electrically orthogonal MC-OQAM subbands, which are generated by the same DAC. Then the cascaded optical couplers are used to combining all subbands and an OBM-OQAM superchannel is thus generated. The subbands are equally spaced at 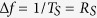, where 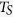 is the symbol period and 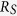 is the symbol rate. The generation of electrical MC-OQAM signal is illustrated in Fig. 2(b). 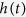 is the impulse response of the pulse shaping filter. Half-symbol delay is introduced by 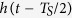. By multiplying 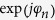, where 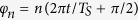, the subband is up-converted and the half-symbol delay alternate between the in-phase and the quadrature components in even and odd subbands.

In Fig. 2(a), we denote the symbols of *nth* subband of *mth* brunch as 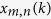,


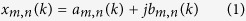


The output waveform is written as


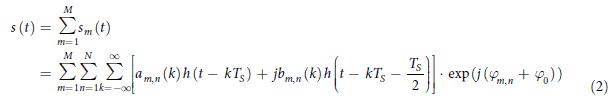


where


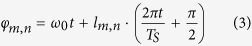



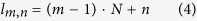


Equation (2) indicates that the signal data between different brunches should be clock-synchronized, and the subcarriers of the superchannel should have the same initial phase 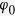. In practical application, to guarantee that each optical carrier has the same initial phase after parallel modulation, a phase shifter can be applied in each brunch, which is shown in Fig. 2(a). Similar structures are also used in coherent WDM^17^, all-optical OFDM^11^, and integrated parallel modulators^18^,^19^ to provide parallel modulation.

In practical application, each optical carrier should correspond to one independent modulator. In (2), *N* is the number of the subbands for one electrical MC-OQAM signal. The bandwidth of an electrical MC-OQAM signal is limited by the bandwidth of the modulator and the corresponding DAC. In the transmitter side, *N* × *M* subbands are generated by one optical comb and *M* modulators. The number of modulators is much less than conventional OFDM-WDM scheme or Nyquist-WDM scheme. For the OBM-OQAM superchannel, the symbol rate 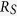 of each subband should be equal for orthogonality. Therefore, the superchannel bandwidth is 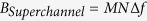, where 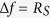
indicates the granularity determined by the user requirement. If 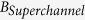 is kept unchanged, larger bandwidth 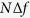 of the electrical MC-OQAM signal means less modulators. In general, the generation procedure of the electrical MC-OQAM signal is realized with digital signal processing (DSP). Within the hardware capability of the transmitter, we can easily change the subbands amount *N* and the subband symbol rate 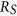 by reconfiguring the DSP algorithm. Therefore, the OBM-OQAM superchannel exhibits an advantage of offering subbands with variable amount and symbol rate while without changing the system configuration, which makes the system flexible and cost effective.

**Figure 3:**
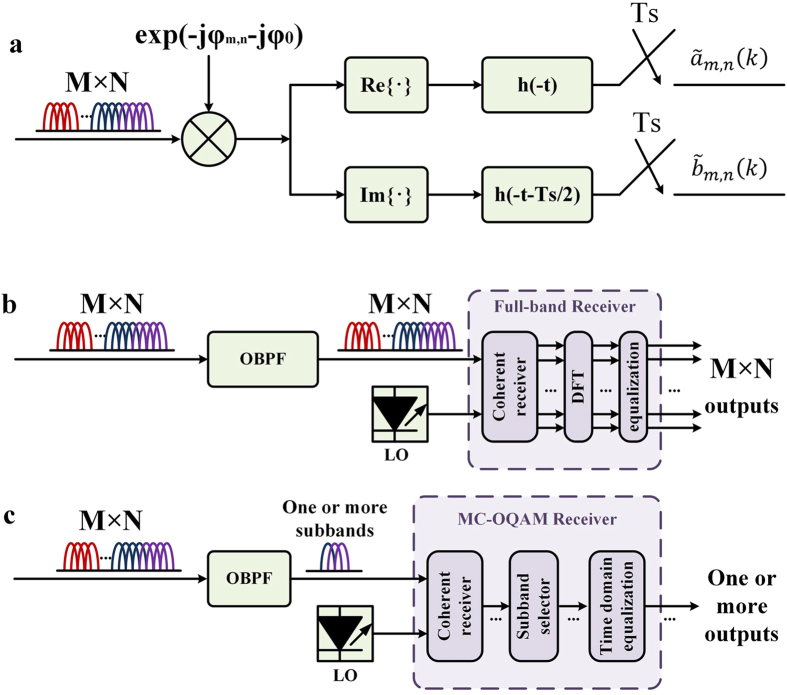
OBM-OQAM receiver scheme. (**a**) Receiving principle. Since the subbands are orthogonal to each other, we can use a down-convertor and a matched filter to demodulate the signal. Two detection scheme: (**b**) full-band detection: Treat the superchannel as an OFDM/OQAM signal and perform full-band receiving with the help of FFT module and frequency domain equalization; and (**c**) detection of only one or several subbands with conventional MC-OQAM receiver.

Figure 3 shows the receiver scheme. At the receiver, the OBM-OQAM signal is demodulated and fed into the corresponding matched filters as shown in Fig. 3(a). Then the filtered signal is sampled at the rate of 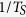. The output symbols of the *nth* subband in the *mth* brunch 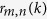 are given as


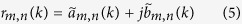


Here 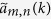 and 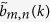 are the real and the imaginary parts of the kth received symbol, respectively. Thus we have


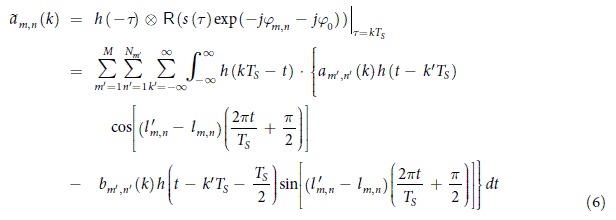


and


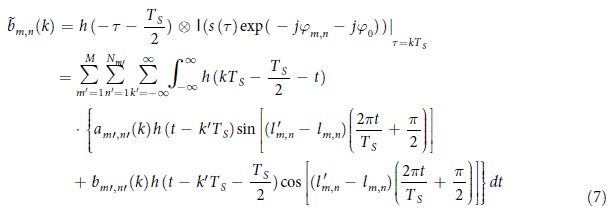


If 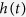 is chosen such that the equalities in equation (9)–(12), 30, 31, 32 are satisfied, there will be:


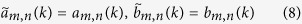


Note that the OBM-OQAM waveform expression (2) and the receiving principle are equivalent to the OFDM/OQAM signal^20^,^21^, the practical superchannel signal can be detected by an OFDM/OQAM receiver as shown in Fig. 3(b). We can also detect one or multiple subbands by employing single-band MC-OQAM receiving algorithm on each subband as shown in Fig. 3(c). Therefore, if the receiver bandwidth can cover the range from one subband to the whole superchannel, any subband can be demodulated individually or jointly with others as a whole.


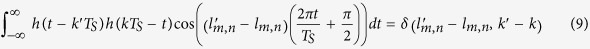



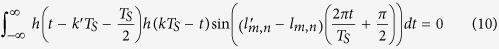



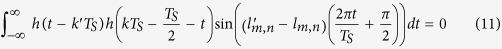



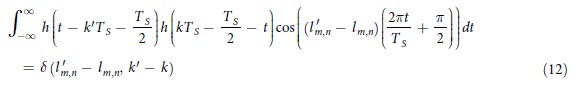


### Proof-of-concept experiment

**Figure 4:**
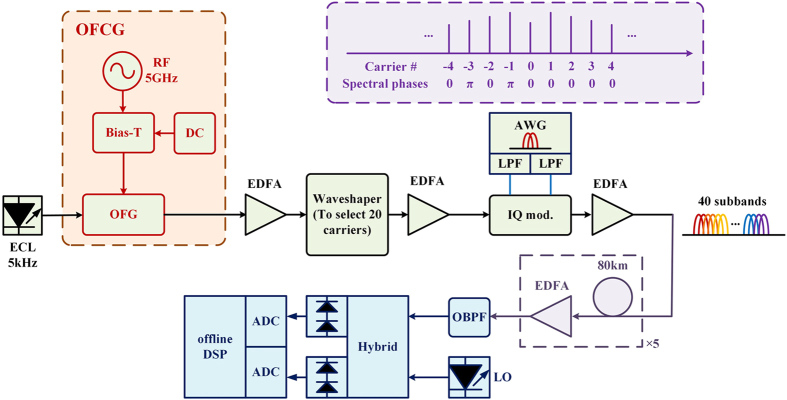
Experimental setup of OBM-OQAM superchannel generation, transmission, and detection. (ECL: external cavity laser; OFCG: optical frequency comb generator; OFG: optical frequency generator; RF: radio frequency; IQ mod.: in-phase and quadrature modulator; AWG: Arbitrary waveform generator; LPF: low pass filter; OBPF: optical band pass filter; LO: local oscillator; ADC: analog to digital convertor).

**Figure 5:**
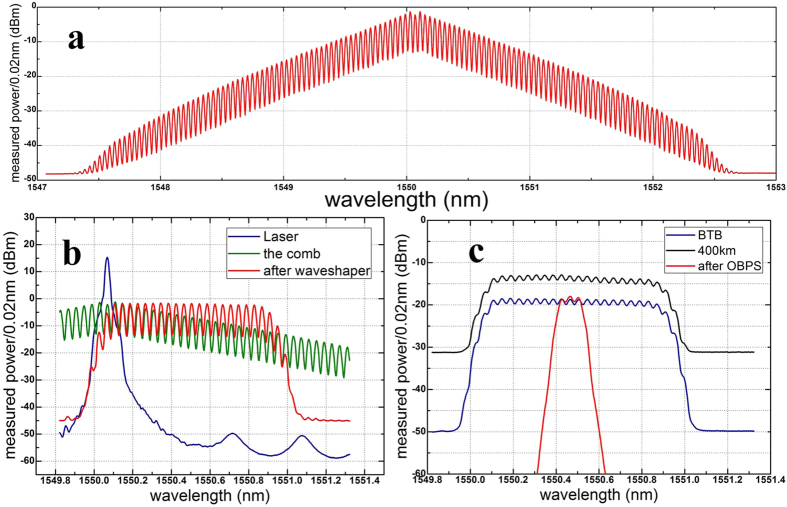
Optical spectra. (**a**) Optical spectra of the comb. (**b**) Optical spectra of the ECL (blue line), part of the comb (green line), and the chosen 20 carriers (red line). (**c**) Optical spectra of the superchannel of back-to-back case (blue line), 400 km SSMF transmission case (black line), and the filtered signal (red line).

A proof-of-concept experiment is demonstrated in this section. Figure 4 shows the generation, transmission, and coherent detection of an OBM-OQAM superchannel. The OFCG, realized by coating high-reflection films on the facets of a LiNbO_3_ phase modulator^22^,^23^, is modulated by 5 GHz Radio Frequency (RF) signal to generate a smooth, broad, stable, and phase-known frequency comb as shown in Fig. 5(a). The insert of Fig. 4 illustrates the phase distribution of the comb according to ref. 23. The right half of the comb has the same initial phase, which is suitable for OQAM signals. After the OFCG, a Finisar waveshaper is used to select carrier No. 1–20 and equalize their optical power as shown in Fig. 5(b). An external cavity laser (ECL) with narrow linewidth of ~5 kHz is used as the light source of the OFCG. An arbitrary waveform generator (Tektronix AWG7122B) operating at 10GSample/s generates two uncorrelated 2.5GBaud electrical MC-OQAM subbands. RRC filters with a roll-off factor of 0.5 are chosen for Nyquist pulse shaping. Analog electric low-pass filters (ELPF) with 3 dB bandwidth of 4.4 GHz are used as anti-aliasing filtering. Each carrier is modulated by two uncorrelated MC-OQAM subbands through the IQ modulator. Thus a single polarization OBM-OQAM superchannel with 100 GHz bandwidth, 40 subbands, and 400 Gbps data rate is generated. The electrical data has half-symbol delay alternating between the in-phase and the quadrature components in even and odd subbands. Therefore the adjacent subbands are orthogonal even if they come from different optical carriers. The transmission link consists of five spans of ~80 km SSMF with Erbium-Doped Fiber Amplifier (EDFA) only amplification. No inline chromatic dispersion compensation is used. For simplification, we use a single IQ modulator to substitute the parallel modulators structure as shown in Fig. 2(a). Since the data of adjacent subbands in the superchannel keep unrelated, this simplification does not influence the conclusion in this paper.

Figure 5(c) shows the spectra of the signal. The blue line is the back-to-back (BTB) case. The black line shows the spectrum after 400 km SSMF transmission. The red line shows the spectrum after 400 km transmission and optical filtering. The resolution is set as 0.02 nm to see spectrum details of the subbands. 40 subbands are spaced equidistantly. However, their spectra are not flat due to the electronic bandwidth limitation of the AWG. Each ‘peak’ of the spectra in Fig. 5(c) contains one optical carrier which is modulated by 2 subbands. The superchannel occupies 100 GHz bandwidth, which is too large for full-band detection with the coherent receiver used in our experiment. Therefore we adopt the receiving scheme in Fig. 3(c) by detecting one subband individually. At the receiver, an OBPF is used as the receiving filter to select the demodulated signal band, which is shown in Fig. 4. Due to the overlapping of the subbands, the adjacent subbands will be partially included. The coherent receiver consists of a 90° optical hybrid, an optical local oscillator (LO) with ~100 kHz linewidth and two pairs of balanced detectors (BD). A real-time sampling oscilloscope (Tektronix DPO72004B) operating at 50 GSample/s stores the electrical waveforms for offline processing.

**Figure 6:**
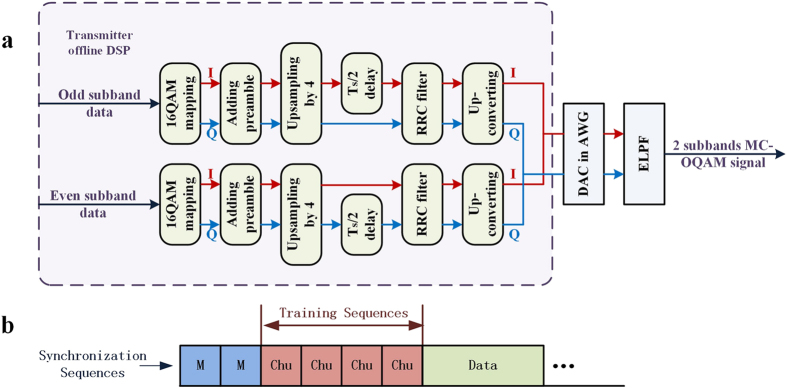
(**a**) Transmitter offline DSP. (**b**) Frame structure of the signal.

Figure 6(a) illustrates the flowchart of the transmitter DSP. At the transmitter, the information data is firstly mapped into 16QAM format and then packed into signal frames. After upsampling by 2 × m, where m is the amount of subbbands generated by AWG, the data is half-symbol delayed alternating between the in-phase and the quadrature components in even and odd subbands. RRC filters are used for pulse shaping. Up-convertors are used to space the subbands at symbol rate interval in frequency domain. Figure 6(b) shows the frame structure of the data. Two 255-bit M-sequences are used for synchronization. Since the training sequences (TS) should be uncorrelated to synchronization sequences, we use Four N-bit Chu sequences *Chu* (*n*) for training. Here *Chu* (*n*) = exp(*j*π*n*^2^/*N*), *n* = 1, 2, ⋯ *N*, and *N* = 256 is adopted in the experiment.

**Figure 7:**
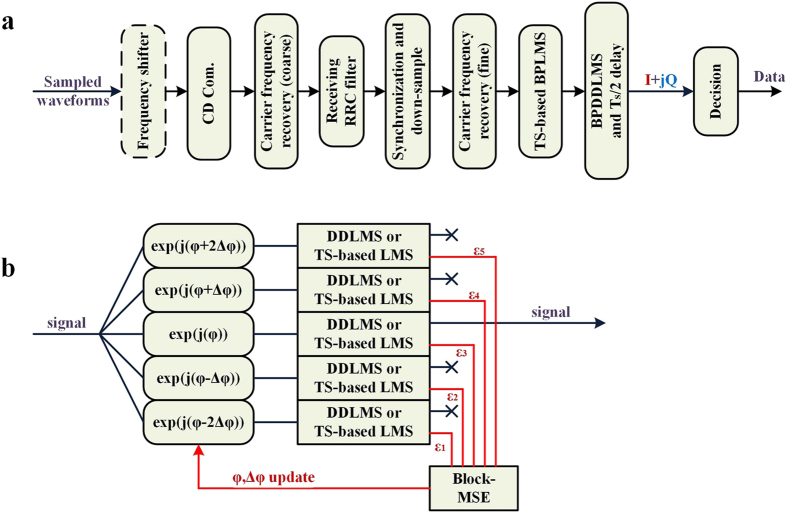
(**a**) Receiver offline DSP. (**b**) Blind phase Decision-Directed Least-Mean-Square (BPDDLMS) and TS-based blind phase LMS (BPLMS) algorithms in OQAM system.

Figure 7 shows the flowchart of the receiver DSP. An optional frequency shifter is firstly used to select the received subband among the optically filtered ones. Then chromatic dispersion (CD) compensation, carrier frequency recovery, matched receiver filtering, header synchronization and down sampling are conducted. Note that the phase noise degrades the channel estimation^13^, and in turn the phase noise removal on the un-equalized signal is difficult. In the DSP we use a novel structure to perform self-adaptively phase noise tracking before equalization, which is shown in Fig. 7(b). The signal is split into 5 copies, which are phase rotated by φ + 2Δφ, φ + Δφ, φ, φ-Δφ, and φ-2Δφ, respectively. Then decision-directed least-mean-square (DDLMS) algorithm or TS-based least-mean-square (LMS) algorithm is done in each brunch. 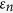 is the constellation error calculated in the decision-directed mode or the TS-aided mode. After each block of *M* symbols, the blockwise mean square error (MSE) is calculated. Then φ and Δφ are updated self-adaptively according to the blockwise MSE result to track the phase noise. In the experiment, we use*M* = 8, and initialize φ = 0°, Δφ = 10°.

**Figure 8:**
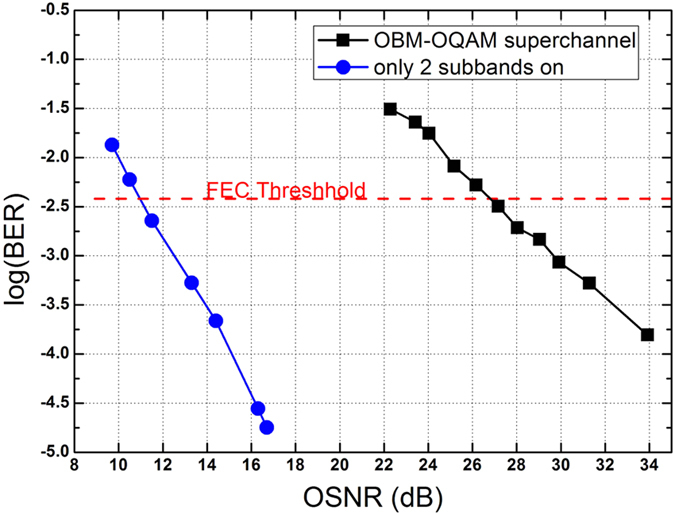
Back-to-back BER performance.

Figure 8 shows the back-to-back BER performance of the superchannel as a function of optical SNR (OSNR) at 0.1 nm resolution. By removing the OFCG and the waveshaper, we get the original 2 subbands. The required OSNR of 40 subbands is ideally 13 dB larger than that of 2 subband (10 · lg(40(*subbands*)/2(*subbands*)) = 13). In our experiment, the measured OSNR requirement of the superchannel at the BER of 3.8 × 10^−3^ is 27 dB, which is 16 dB larger than the original 2 subbands, indicating that a 100 GHz OBM-OQAM/16QAM superchannel has about 3 dB penalty for subbands aggregation.

**Figure 9:**
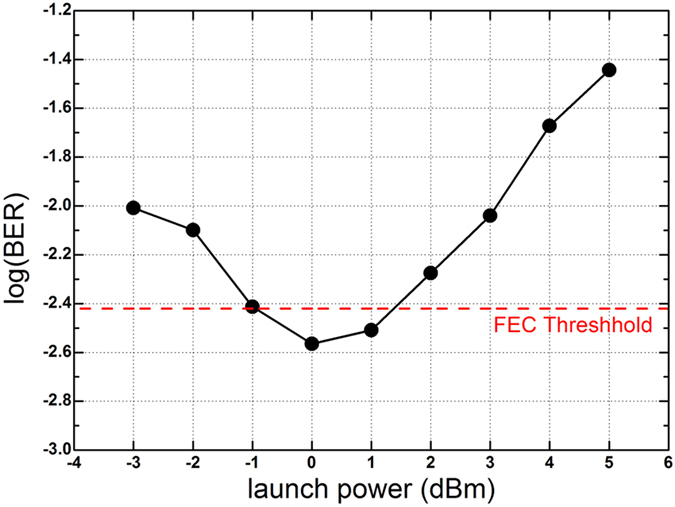
Measured BER of the 21st subband over 400 km SSMF.

**Figure 10:**
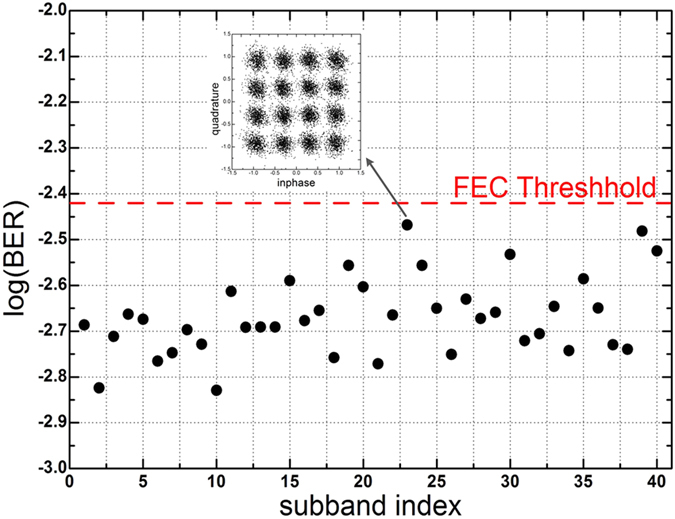
Measured BER for OBM-OQAM superchannel over 400 km SSMF.

Figure 9 shows the BER of the 21st subband versus launch power of the superchannel over 400 km SSMF. The optimal launch power is 0 dBm. Figure 10 shows the measured BER results of all 40 subbands after 400 km SSMF transmission with the optimal launch power. The inset is the constellation of the 23rd subband, which has the worst performance. The BER of each subband is lower than 3.8 × 10^−3^, which is the threshold for 7% forward error correction (FEC) overhead. The BER of each subband is measured based on 4 × 10^5^ data bits. The average BER for the entire superchannel is 2.1 × 10^−3^.

## Discussion

Compared with Nyquist-WDM, OFDM-WDM and OBM-OFDM techniques, the OBM-OQAM superchannel does not need guard bands between subbands because they overlap without resulting in crosstalk even if they come from different carriers. According to the theoretical model explained in the paper, we provide two alternative receiving schemes: detecting the full-band superchannel by using the OFDM/OQAM procedure, or detecting one or more subbands by employing single-band MC-OQAM algorithm on each subband separately. With these two schemes, any subband within the receiver wavelength range can be demodulated individually or together with others.

Thanks to the OFCG and a narrow linewidth ECL, a smooth, broad, stable, phase-known, low phase noise frequency comb is generated, which is the key element to our high speed OBM-OQAM transmission system. In the experiment, we generate a 400 GHz, 4 b/s/Hz, single-polarization 16QAM OBM-OQAM superchannel which occupies 0.8 nm bandwidth and contains 40 subbands. In the receiver offline DSP, the phase noise recovery needs to be done before channel compensation. Therefore, we apply the TS-based blind phase least-mean-square (BPLMS) and the self-adaptive blind phase decision-directed least-mean-square (BPDDLMS) algorithms, in which the self-adaptively phase noise recovery is conducted before time domain equalization. The experiment results show that, the OBM-OQAM signal has about 3 dB penalty in subband aggregation. After transmission over 400 km, the BER of each subband is lower than 3.8 × 10^−3^, which is the threshold for 7% FEC overhead.

In conclusion, we propose for the first time the OBM-OQAM superchannel and its generation and detection scheme. OBM-OQAM superchannel has the advantages of large capacity, high spectral efficiency, and especially, high flexibility since it can offer subbands of variable symbol rate without changing the system configuration.

## Methods

### Receiver offline DSP details

The receiver offline DSP is shown in Fig. 7a. The accumulated CD is roughly compensated by an FIR filter. Then the carrier frequency is coarsely recovered by a phase increment estimation algorithm well known in wireless communication^24^. A matched receiving RRC filter is adopted to satisfy the Nyquist first criterion. Synchronization is conducted by searching for the correlation peak between the known synchronization sequences and the signal. Then the signal is down-sampled to 2 samples per symbol. The fine carrier frequency recovery is conducted by calculating the average phase increment of the training sequences. The train sequences are also used for the TS-based LMS algorithm. ref. 16 applied phase tracking in the equalization. Considering the laser linewidth in the transmitter may be unknown to the receiver in practical networks, the receiver DSP should be conducted along with unknown transmitter laser linewidth. Comparing with ref. 16, our BPDDLMS and TS-based BPLMS algorithms use 5 equalization brunches rather than 3, making the self-adaptively phase tracking possible. In Δφ updating, if 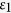 or 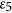 is the minimum, Δφ will be magnified; conversely, if 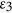 is the minimum, Δφ will be minified. Consequently, the tracking phase angle of kth block 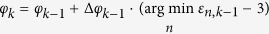. In the DDLMS and the TS-based LMS algorithms, the linear equalizer is *T*_*S*_/2 spaced, the received constellation is obtained according to 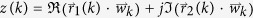, where 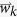 is the equalizer weights^13^; 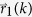 and 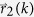 are the signal samples vectors for kth symbol starting with an even and an odd sample, respectively. The equalizer weights 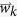 are updated on 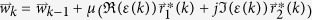, where μ is the update coefficient.

## Additional Information

**How to cite this article**: Zheng, Z. *et al.* Orthogonal-band-multiplexed offset-QAM optical superchannel generation and coherent detection. *Sci. Rep.* **5**, 17891; doi: 10.1038/srep17891 (2015).

## Figures and Tables

**Figure 1:**
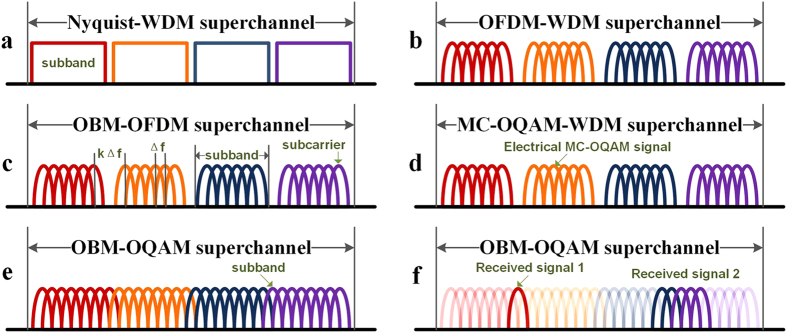
Scheme of optical superchannel (a) Nyquist-WDM superchannel. (**b**) OFDM-WDM superchannel. (**c**) OBM-OFDM superchannel. (**d**) MC-OQAM-WDM superchannel. (**e**) OBM-OQAM superchannel. (**f**) Examples of the received OBM-OQAM signal.
